# Bridging the vertical mismatch: elevation-dependent drivers and conservation strategies for mountain biodiversity

**DOI:** 10.1093/nsr/nwag163

**Published:** 2026-03-14

**Authors:** Tao Hu, Yi’na Hu, Jiayao Shu, Zihan Xu, Qiancheng Lv, Jianquan Dong, Yanxu Liu, Jian Peng

**Affiliations:** Laboratory for Earth Surface Processes, Ministry of Education, College of Urban and Environmental Sciences, Peking University, China; School of Environmental and Geographical Sciences, Shanghai Normal University, China; Laboratory for Earth Surface Processes, Ministry of Education, College of Urban and Environmental Sciences, Peking University, China; School of Soil and Water Conservation, Beijing Forestry University, China; Laboratory for Earth Surface Processes, Ministry of Education, College of Urban and Environmental Sciences, Peking University, China; College of Landscape Architecture, Beijing Forestry University, China; State Key Laboratory of Earth Surface Processes and Disaster Risk Reduction, Faculty of Geographical Science, Beijing Normal University, China; Laboratory for Earth Surface Processes, Ministry of Education, College of Urban and Environmental Sciences, Peking University, China

The Kunming-Montreal Global Biodiversity Framework (KM-GBF) represents a global commitment to halt biodiversity loss, mainly through its target to conserve 30% of land by 2030. Mountains are often regarded as natural beneficiaries of this agenda: their remoteness, steep terrain and harsh climates have historically been thought to limit human activities, positioning them as refugia for biodiversity under accelerating global change [1]. While the KM-GBF is ambitious in spatial coverage, its area-based targets and indicators are predominantly framed in horizontal terms, offering limited insights into how human pressures and outcomes of biodiversity conservation are distributed along elevation gradients in mountain regions [2, 3]. Consequently, biodiversity conservation success may be declared simply because area targets are met, potentially obscuring the fact that biodiversity and ecological functions continue to degrade across critical elevation gradients.

Elevation is not merely a geographical variable. In mountain ecosystems, it constrains ecological processes and restructures human activities [4], shaping the elevational decoupling among human pressure, biodiversity loss and the implementation of protected areas (Fig. 1a). Importantly, this vertical dimension does not imply an additional conservation target or a revision of the KM-GBF itself. Rather, it represents an analytical lens through which the effectiveness of area-based conservation can be more accurately assessed in vertically heterogeneous landscapes. When human pressure, biodiversity loss and protected area coverage are vertically misaligned in mountains, conservation outcomes may fail even though global area-based targets are nominally met, which is increasingly common in mountain regions worldwide [5]. As a result, recognizing this vertical mismatch is essential to effectively achieve area-based conservation targets in mountain regions.

**Figure 1.**
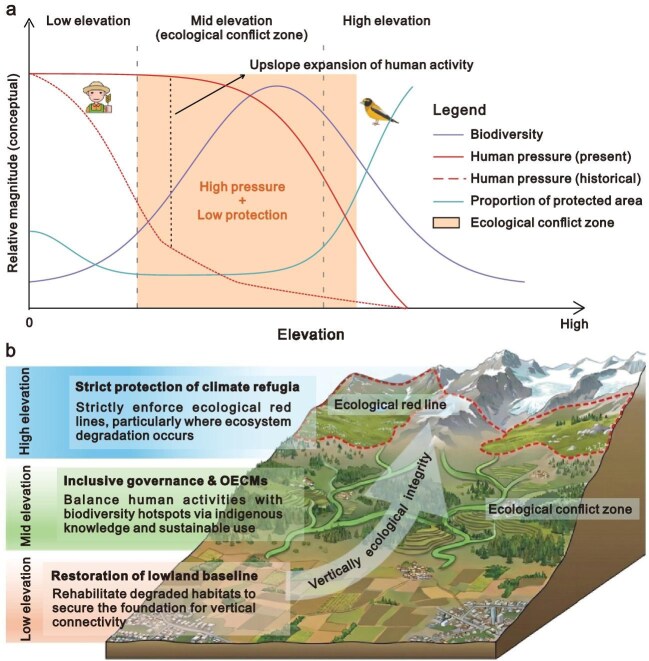
Elevation-dependent ecological conflict and stratified conservation strategies in mountain regions. (a) Conceptual vertical mismatch illustrates mechanism of ecological conflict in mountain regions; (b) Elevation-stratified management framework for safeguarding mountain biodiversity, with the background image AI-generated and included for visual design only. The X-axis represents elevation. The Y-axis shows the relative magnitude of three key variables normalized for conceptual comparison. Ecological conflict zone: high human pressure structurally converges with peak biodiversity richness, precisely where current proportion of protected areas is at its lowest. Historically, human pressure (red dashed line) was confined to lowlands. Currently, human activities are expanding upslope, resulting in the emerging crisis at mid elevations. Other Effective Area-Based Conservation Measures (OECMs) is a geographically defined area other than a Protected Area, which is governed and managed in ways that achieve positive and sustained long-term outcomes for the *in-situ* conservation of biodiversity, with associated ecosystem services and socio-economic values.

## ECOLOGICAL CONFLICT AT MID ELEVATIONS

*Classical view vs. new reality.* The classical view assumes that human pressure declines with elevation, framing lowland as zones of intensive human use and high mountains as largely natural spaces. This assumption has served as a foundation for land-use planning, the designation of protected areas and global assessments of human footprint for decades [4]. However, this situation is undergoing significant transformation. Global warming is alleviating thermal constraints on agriculture and human settlement in mountain regions, while lowland land resources in many regions are increasingly overexploited. Human activities are no longer restricted to valley bottoms but are gradually shifting upslope, altering long-established relationships between elevation, land use and ecological integrity [6]. Crucially, this upslope expansion of human activities carries a risk of irreversibility. Once human pressure exceeds critical elevational thresholds, the resulting biodiversity loss is difficult to reverse due to the inherently slow recovery rates of high-altitude ecosystems.

*Ecological conflict zone.* While some studies have reported linear relationships between species richness and elevation, mountain biodiversity more frequently exhibits a unimodal pattern at broader spatial scales, with species richness peaking at mid elevations. Previous studies also found that range-size rarity hotspots in mountain regions spanned a broad elevation gradient, with high values located at mid elevations [7]. These zones host a variety of climatic niches and habitat diversity, making them disproportionately important for biodiversity conservation. Concurrently, mid elevations are becoming more accessible to human activities, situated at the boundary between densely populated lowlands and the less populated high mountains [4]. Intense human pressure extends well into mid elevations. Thus, mid elevations are no longer transitional gradients but are evolving into ecological conflict zones, where the expansion of human activity is intersected with the unimodal peak of biodiversity. The squeeze on mid elevations also represents a structural risk for mountain ecosystems because they link the ecological processes of lowland and high elevations [6]. Landscape fragmentation and anthropogenic pressure in these ranges can interrupt species migration pathways along elevation gradients and diminish the buffering capacity of mountain regions against climate and land-use change. Ecological recovery rates typically decline with increasing elevation, while species occupying mid elevations have more limited ranges [8]. Consequently, equivalent levels of disturbance can make larger and more persistent biodiversity loss in these zones compared with lowlands.

*Intensification drivers.* Multiple drivers are intensifying pressures at mid elevations in global mountain regions. First, although biodiversity conservation policies have increasingly restricted new infrastructure development in many regions, historical construction of road and built-up land has reduced spatial isolation from human activities and increased accessibility to previously remote areas [9]. Second, the upward

shift of crop cultivation and the expansion of tourism infrastructure are advancing into these zones, driven by market demand, climate suitability and development incentives [6]. Third, spatial planning fails to keep pace with these swift land-use transformations, while conservation measures remain insufficient or poorly implemented in many mountain regions [2]. As a result, the mid elevations became zones where land-use intensification, biodiversity concentration and governance gaps overlap, amplifying ecological vulnerability. The consequences of this squeeze extend beyond biodiversity loss alone. Degradation occurring at mid elevations adversely affect mountain communities by decreasing nature’s contributions to people [10]. Many essentials of nature’s contributions, including regulation of freshwater quantity, location and timing, regulation of hazards and extreme events, and medicinal, biochemical and genetic resources, are disproportionately generated or lost at these elevations. As ecological integrity declines, communities face higher risks of droughts, floods and landslides, and their traditional ways of life, once perfectly suited to the mountain context, are now under threat.

*Mismatched conservation.* Current conservation strategies remain vertically misaligned, particularly at mid elevations. Protected areas in mountain regions are disproportionately situated at very high elevations [2], often dominated by rock, ice or sparsely vegetated terrain. Although these regions play important roles in regulating hydrological processes and provision of natural habitat, their preferential protection is frequently associated with lower opportunity costs and fewer conflicts with economic development [11,12]. Conservation efforts may be skewed toward high elevations, while ecologically rich mid elevations remain comparatively underrepresented. This pattern reflects not the shortcoming of area-based targets, but an implementation bias in how conservation measures are spatially allocated in mountainous landscapes. Even as protected area coverage expands under the KM-GBF, mountain conservation strategies often fail to account for how biodiversity loss varies with elevation. Measures such as the establishment of Other Effective Area-based Conservation Measures (OECMs) remain underdeveloped across these zones. OECMs are geographically defined areas outside formal protected areas that are governed and managed to achieve sustained, long-term *in situ* biodiversity conservation, along with associated ecosystem services and socio-economic values. Roughly half of global mountains fail to meet the 17% conservation target at any elevation, and most cannot meet this target across large portions of their elevation gradient [2]. For example, priority gaps of biodiversity conservation in mountain regions occur at low to mid elevations in Africa and Asia, at both low and high elevations in Europe, at mid to high elevations in Oceania, and across nearly all elevations in South America [2]. Such vertical gaps lead to spatially successful but functionally misdirected conservation efforts, safeguarding areas of low pressure while leaving mid elevations insufficiently protected. By prioritizing horizontal area coverage without considering elevation-dependent dynamics, current frameworks will overestimate conservation effectiveness in mountain regions, allowing biodiversity loss and ecosystem degradation to continue largely unnoticed in mid elevations.

## STRATEGIES FOR BRIDGING THE VERTICAL GAP

*From horizontal targets to vertically ecological integrity.* Effective mountain conservation must shift focus from horizontal area coverage to ensuring vertically ecological integrity (Fig. 1b). Rather than treating protected areas as isolated polygons, conservation planning should prioritize elevational connectivity, ensuring structural and functional linkages from lowlands to high elevations [2,13]. This shift is critical for facilitating species’ upward migration under the context of global warming. However, safeguarding vertical integrity requires more than simply expanding the elevation range coverage of protected areas. Different elevation zones play distinct ecological roles and therefore demand differentiated management priorities [13]. To improve vertically ecological integrity, conservation planning should incorporate elevational connectivity metrics such as habitat connectivity and barrier permeability into systematic planning frameworks, using tools like least-cost corridor modeling to assess functional linkages across elevation zones rather than only across horizontal space. Conservation metrics should move beyond total surface area or simple elevation coverage to evaluate whether conservation efforts are functionally aligned with elevation-specific ecological processes and vertically connected across elevation gradients. This shift aligns with nature-based solutions, emphasizing that the resilience of mountain regions depends not just on how much is protected, but on preserving the functional linkages between lowland pressure zones and high-altitude refugia. Management priorities should differ across elevation zones. Low elevations should focus on restoring degraded ecosystems to re-establish the ecological baseline of mountain regions. Mid elevations require adaptive and multifunctional governance, often through flexible measures such as the establishment of OECMs, to maintain vertical corridors while accommodating sustainable land use. High elevations should prioritize the strict protection of slow-recovering ecosystems and climate refugia, with clear thresholds to prevent damage [8].

*Elevation-based thresholds for biodiversity conservation.* Management policies must recognize that ecological recovery rates are uneven across elevations, with slower recovery at higher altitudes [8]. Consequently, applying uniform environmental regulations across elevations is unsuitable and ineffective. We propose the implementation of elevation-based thresholds to safeguard these climate refugia [14]. Environmental impact assessments and policy frameworks must incorporate elevation-based indicators, rather than relying on protected area coverage that can overlook severe degradation at mid elevations. Indicators such as infrastructure density (e.g. road length per km^2^) and land-use conservation proportion must be used to restrict socio-economic development in critical zones. Policy frameworks such as Colombia’s Páramo Law and the Alpine Convention have successfully identified ecological thresholds, and translated them into spatial zoning. Through legally delimiting high-altitude ecosystems based on specific environmental transitions, these frameworks demonstrate that regulating development via vertical boundaries is achievable. The Global Observation Research Initiative in Alpine Environments (GLORIA) serves as the essential scientific foundation for defining the threshold. With over 120 standardized monitoring sites, GLORIA enables the rigorous detection of these critical tipping points across diverse mountain regions. Its ability to identify elevations where vegetation recovery drops or functional diversity declines provides policymakers with measurable indicators to set clear spatial and elevational limits for conservation. Expanding the monitoring network to add more sites in underrepresented regions such as mid elevations would further strengthen our capacity to guide conservation decisions.

*Inclusive governance and OECMs for the ecological conflict zone.* In the ecological conflict zone where expanding human activities structurally converge with biodiversity hotspots, strict protection is often impractical due to long-standing local livelihoods and thus OECMs are advocated. This inclusive governance limits high-intensity development (e.g. mining and large dams) while allowing sustainable livelihood activities such as ecotourism. In China, the management of Yulong Snow Mountain Community OECMs have integrated biodiversity monitoring with local management and traditional grazing practices, demonstrating how communities can protect species without strictly isolating the area [5,15]. Internationally, Canada’s Central Purcell Mountains Indigenous Protected and Conserved Area showed that indigenous-led stewardship in mountain regions could achieve conservation goals while supporting traditional agricultural land use. Integrating local governance systems and place-based knowledge into national conservation planning offers an effective pathway to manage ecological conflict. To implement OECMs in mountain regions, clear biodiversity performance criteria must be established. Areas should be qualified as OECMs if they maintain key indicators such as species richness, habitat connectivity across elevation zones, or specific ecological functions relative to reference conditions. Many mountain regions are already governed by indigenous peoples and local communities. Their place-based knowledge and institutions are well adapted to vertical heterogeneity and seasonal dynamics, making them well suited to monitor and sustain these criteria [15]. Integrating these criteria and local monitoring efforts into national biodiversity reporting systems and national protected area registries ensures that OECMs contribute substantively to global biodiversity targets.
